# Assessment of sclerostin levels in the gingival crevicular fluid of patients with periodontitis: A clinico-biochemical crosssectional study

**DOI:** 10.34172/japid.2023.009

**Published:** 2023-05-27

**Authors:** Nisha Ashifa, Krishnan Viswanathan, Sivapragasam Srinivasan, Senthil kumar, Rajasekar Sundaram, Varsha k. Pavithran

**Affiliations:** ^1^Department of Periodontology, Rajah Muthiah Dental College & Hospital, Annamalai University, Chidambaram, Tamil Nadu, India; ^2^Department of Public Health Dentistry, Rajah Muthiah Dental College & Hospital, Annamalai University, Chidambaram, Tamil Nadu, India

**Keywords:** Alveolar bone loss, ELISA, Gingival crevicular fluid, Periodontitis, Sclerostin

## Abstract

**Background.:**

Sclerostin, a glycoprotein, plays a key role in regulating bone mass. In this study, sclerostin levels in the gingival crevicular fluid (GCF) were assessed in patients with Stage III Grade C generalized periodontitis (SIII-GC) and Stage III Grade B generalized periodontitis (SIII-GB).

**Methods.:**

This cross-sectional study included 30 participants divided equally into three groups: group I (gingival health), group II (SIII-GC), and group III (SIII-GB). Clinical periodontal parameters like plaque index (PI), gingival bleeding index (GBI), probing pocket depth (PD), and clinical attachment level (CAL) were recorded. A sandwich ELISA was used to determine the sclerostin levels in GCF samples. One-way ANOVA and post hoc Tukey tests were used to analyze the clinical parameters and GCF sclerostin levels. The association between GCF sclerostin levels and periodontal parameters was assessed using Pearson’s correlation coefficient (r).

**Results.:**

Patients in groups II and III had much higher sclerostin levels in their GCF than in group I (*P*≤0.05). In contrast, no significant difference in sclerostin levels was observed between the two diseased conditions (*P*=0.841). Concerning periodontal parameters, a statistically significant difference was observed between the three groups. There was a positive correlation between the periodontal clinical parameters and the expression levels of sclerostin in GCF (*P*≤0.05).

**Conclusion.:**

Increased expression of sclerostin in GCF in patients with periodontitis indicated that it could be considered a reliable biomarker of periodontal disease activity.

## Introduction

 Periodontal disease is a common chronic inflammatory condition that occurs when there is a disturbance in the interplay between the host immune reaction and periodontal pathogens.^1^ This disturbance progresses to cause periodontal attachment loss and alveolar bone resorption.^2^ The 2017 World Workshop on the Classification of Periodontal and Peri-Implant Diseases and Conditions has categorized periodontitis into different stages and grades. Staging gives us a vivid picture of the extent, severity, and complexity of the management of periodontitis, while grading helps us determine the disease progression rate, estimate future risks and prognosis, and identify the impact of systemic illness on periodontal disease.^3^

 Biomarker research in periodontology aims to pave the way for high-impact diagnostics, which can considerably enhance clinical diagnosis and treatment plans, patient education and acceptance, and health care finances.^4^ Several substances present in the body and body fluids have been employed as biomarkers to assess periodontal disease. The biological fluids that can be used as a source for periodontal biomarkers are blood, serum, plasma, saliva, and gingival crevicular fluid (GCF).^5^ Compared to other sources, GCF provides more precise, sensitive, and reproducible data on a cellular response that is specific to the periodontal disease site and can be used to forecast further periodontal deterioration.^1,2,5^

 A 190-residue glycoprotein called sclerostin is encoded by the SOST gene, which is located on the long arm of chromosome 17.^6-8^ Sclerostin modulates bone mass by inhibiting osteoblastic cell proliferation, differentiation, and mineralization.^9,10^ It decreases the viability of osteoblasts and osteocytes, creating a discrepancy in bone turnover and favoring bone resorption.^11,12^ It acts as a bone morphogenic protein (BMP) antagonist and a Wingless-related integration site (Wnt) signaling antagonist.^10,13^ Sclerostin binds to low-density lipoprotein receptor protein (LRP) 5/LRP6 complex, thus blocking Wnt binding and Wnt/β-catenin signaling.^14-16^

 The significance of sclerostin in bone metabolism has been clarified by recent in vitro and in vivo studies.^1,2,6,11,17-20^ Sclerostin is known to affect periodontal health and disease status. Considering the above biological effects of sclerostin, the present study investigated whether GCF sclerostin levels could be used as a biomarker of periodontal disease activity in patients with Stage III Grade C generalized periodontitis (SIII-GC) and Stage III Grade B generalized periodontitis (SIII-GB).

## Methods

###  Study participants 

 A cross-sectional study was conducted in the Department of Periodontology between June and September 2019. Before beginning the study, Institutional Ethical Committee approved the research protocol and granted Ethical Clearance (IHEC/575/2019). All the outpatients directed to the Department of Periodontology aged 19‒40, both males and females, were screened for this study. For this study, written informed consent was obtained from each participant before participating. This study followed Strengthening the Reporting of Observational Studies in Epidemiology (STROBE) guidelines.^21^

 Participants were chosen and divided into three study groups after a preliminary screening based on the “2017 World Workshop on the Classification of Periodontal and Peri-Implant Diseases and Conditions.”^3,22^

- Group I: gingival health, systemically and periodontally healthy participants with < 10% of bleeding sites and probing depths ≤ 3 mm
- Group II: patients with SIII-GC, interdental clinical attachment loss ≥ 5 mm, probing depth ≥ 6 mm, vertical bone loss ≥ 3 mm involving > 30% of teeth with a rapid rate of progression, without grade modifiers, who were otherwise systemically healthy
- Group III: patients with SIII-GB, with interdental clinical attachment loss ≥ 5 mm, probing depth ≥ 6 mm, vertical bone loss ≥ 3 mm involving > 30% of teeth with a moderate rate of progression, without grade modifiers, who were otherwise systemically healthy.

Individuals with systemic illness, adverse habits, pregnancy, a history of drug therapy, and those who underwent periodontal therapy in the preceding six months were excluded from the study. In addition, this study was controlled for known biases and confounders.

###  Sample size calculation

 Using statistical power analysis G*Power software^23^ and considering F-tests, one-way ANOVA, fixed effects, and omnibus, the total sample size (n) for the current study was estimated at 30 by maintaining

- an α error of 0.05 at 95% CI,
- a β error of 0.05,
- power of the test (1-β error) as 95%,
- number of groups at 3,
- effect size (Cohen’s f statistic) at 0.7977.^2^

To allow for unanticipated deviations from statistical assumptions, out of the 30 patients, 10 patients (n = 10) were equally distributed into three groups, i.e., 10 samples in each group were used for assessing the sclerostin levels in the GCF of patients.

###  Clinical examination

 A detailed case history was acquired from the thirty participants enrolled in the study, following which they were subjected to a thorough periodontal examination.

 The intraclass correlation coefficient (ICC) based on McGraw and Wong’s (1996) Convention for intra-rater reliability at two points in time (48 hours apart) by using a two-way mixed effects model, single rater/measurement type and absolute agreement definition was found to be 0.997 (*P* ≤ 0.05), indicating excellent reliability.^24^

 The following periodontal clinical parameters were recorded: plaque index (PI) (Silness & Loe 1964), gingival bleeding index (GBI) (Ainamo and Bay, 1975), probing pocket depth (PD), and clinical attachment loss (CAL).

###  GCF sample collection

 The GCF samples were harvested from the deepest periodontal pocket (most representative tooth site) from groups II and III and the most convenient tooth site in group I. Gargling with sterile water was initially recommended to remove loose debris from the tooth surfaces. To prevent contamination of the samples, cotton rolls or gauze was used to dry and isolate the test site. GCF samples were collected using standardized paper strips (PerioPaper, Oraflow Inc.). Paper strips were inserted into the gingival crevice and left undisturbed for 30 seconds. Blood or saliva-contaminated strips were rejected. A calibrated device that works on the principle of electronic impedance (Periotron 8000, Oraflow Inc.) was used to calculate the GCF volume. The results from the electronic GCF measuring device were converted into a real volume (microliter) concerning the standard curve. The collected GCF samples were analyzed using a commercially available sandwich Enzyme-Linked Immunosorbent Assay (ELISA) kit (ELABSCIENCE^®^ HUMAN SCLEROSTIN ELISA KIT) according to the manufacturer’s instructions, and the results were statistically evaluated.

###  Statistical analysis

 Data regarding PI, GBI, PD, CAL, and GCF sclerostin level values for three groups were entered into Microsoft Excel and analyzed using IBM SPSS 20 (IBM Corp., Armonk, N.Y., USA). The Shapiro-Wilk test was used to determine the normality of the data, which revealed that it followed a normal distribution. Descriptive statistics were derived as means, standard deviations, and 95% confidence intervals. The PI, GBI, PD, CAL, and GCF sclerostin levels between the three groups were analyzed using one-way ANOVA followed by multiple comparisons with Tukey tests (α = 0.05). Pearson’s correlation coefficient (r) was used to evaluate the association between GCF sclerostin levels and other periodontal parameters. The level of statistical significance was set at *P* ≤ 0.05.

## Results

###  Descriptive statistics and clinical findings

**Figure 1:**
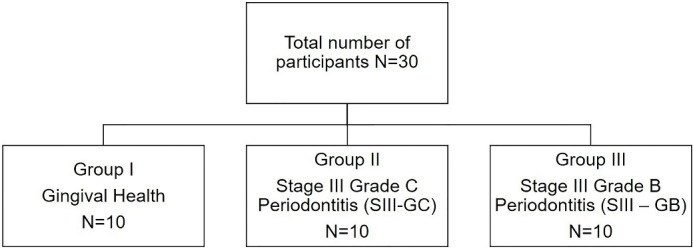
Flowchart of study participants and grouping

 After a thorough assessment based on inclusion and exclusion criteria, 30 patients were included in the study, with 10 participants in each group (Figure 1). The collective demographic data are presented in Table 1. The study population comprised 30 patients (16 males and 14 females) with a mean age of 29.53 ± 5.67 years. The clinical periodontal parameters of the study population are presented in Table 2.

**Table 1:** Descriptive statistics of study participants in control and case groups

**Demographic characteristics (n=30)**		**Mean±SD ** **(Min-Max)**
Age (y)		29.53 ± 5.67 (19-40)
Gender	Male [n (%)]	16 (53.3%)
	Female [n (%)]	14 (46.7%)

**Table 2:** Periodontal parameters of the study population

**Periodontal parameters** **(n=30)**	**Mean±SD**	**Min**	**Max**
PI	0.36 ± 0.27	0.03	1.22
GBI	34.82 ± 23.35	1.70	70
PPD (mm)	4.98 ± 2.30	1.78	8.65
CAL (mm)	5.18 ± 2.50	1.78	9.52
GCF sclerostin level (pg/mL)	302.27 ± 99.34	131	566

PI: plaque index, GBI: gingival bleeding index, PPD: probing pocket depth, CAL: clinical attachment level, GCF: gingival crevicular fluid.

**Figure 2:**
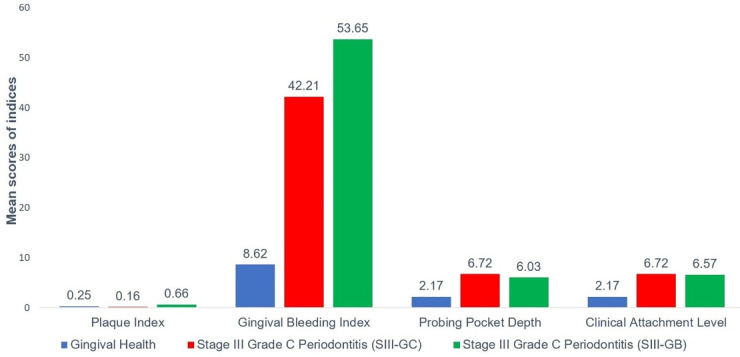
Means of periodontal parameters in the three study groups

 Considering the periodontal clinical parameters (PI, GBI, PD, and CAL), there was a significant difference in the mean value between the three groups (*P* = 0.000) (Table 3 and Figure 2). Intergroup comparisons for the periodontal parameters between the three groups are summarized in Table 3. While comparing the mean value of PI between the three groups, there was a significant mean difference between groups I and III (*P* = 0.000) and groups II and III (*P* = 0.000), with no significant difference between groups I and II (*P* > 0.05). Concerning other periodontal clinical parameters (GBI, PD, and CAL), a significant difference was noted in the mean value between groups I and II and between groups I and III (*P* = 0.000), with no significant difference between groups II and III (*P* > 0.05).

**Table 3:** Comparison of various periodontal parameters between control and case groups

**Periodontal parameters**	**Group I** **(n=10)**	**Group II** **(n=10)**	**Group III** **(n=10)**	***P*** **value** ^c^
PI (mean ± SD)	0.25 ± 0.67	0.16 ± 0.10	0.66 ± 0.27^a,b^	0.000^d^
GBI (mean ± SD)	8.62 ± 5.17	42.21 ± 18.96^a^	53.65 ± 12.42^a^	0.000^d^
PPD (mean ± SD)	2.17 ± 0.30	6.72 ± 1.44^a^	6.03 ± 1.22^a^	0.000^d^
CAL (mean ± SD)	2.17 ± 0.30	6.79 ± 1.52^a^	6.57 ± 1.65^a^	0.000^d^
GCF sclerostin level (mean ± SD)	213.90 ± 44.35	356.35 ± 45.37^a^	336.57 ± 120.59^a^	0.001^d^

PI: plaque index, GBI: gingival bleeding index, PPD: probing pocket depth, CAL: clinical attachment level, GCF: gingival crevicular fluid, NS: Not significant. Comparisons between the groups were performed using one-way ANOVA, followed by post hoc Tukey tests. Post hoc Tukey tests: ^a^Significantly different from group I: gingival health (*P* ≤ 0.05); ^b^Significantly different from group II – SIII-GC (*P* < 0.05). ^c^One-way ANOVA test value; ^d^Statistically significant (*P* < 0.05).

###  Biochemical findings

**Figure 3:**
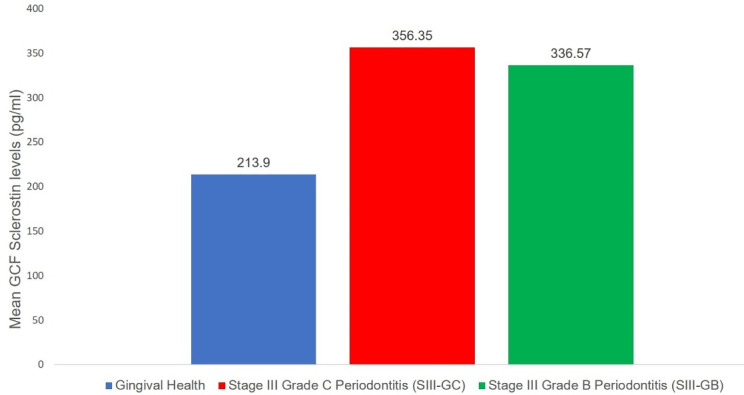
Mean GCF sclerostin levels in the three study groups

 The mean GCF levels of sclerostin were the lowest in group I (213.90 ± 44.35 pg/mL) compared to group II (356.36 ± 45.37 pg/mL) and group III (336.57 ± 120.59 pg/mL), as observed by mean and standard deviation, which was statistically significant (*P* ≤ 0.05) (Table 3 and Figure 3). Intergroup comparison of GCF sclerostin levels between the three groups revealed a significant difference in the mean values of GCF sclerostin levels between groups I and II and between groups I and III (P ≤ 0.05). No difference was observed in mean GCF levels of sclerostin (*P* = 0.841) between the patient groups (groups II and III), indicating that the expression of the glycoprotein was similar in groups II and III (Table 3).

###  Correlations

 Pearson’s correlation (r) between GCF sclerostin levels and clinical periodontal parameters is presented in Table 4. The highest positive correlation was found when PD was associated with GCF sclerostin levels, and this association was significant (*P* = 0.000). Other periodontal parameters (GBI and CAL) had a positive, significant correlation when compared to GCF sclerostin levels (*P* = 000 and *P* = 0.002, respectively). The least positive correlation was found between PI and GCF sclerostin levels, which was not significant (*P* = 0.647). Thus, the sclerostin levels in GCF (pg/mL) increased with an increase in periodontal clinical parameters.

**Table 4:** Correlation of various periodontal parameters with GCF levels of Sclerostin

**Periodontal parameters**	**Pearson’s correlation coefficient (r)**	***P*** ** value**
PI	0.087	0.647
GBI	0.552	0.002*
PPD	0.668	0.000*
CAL	0.651	0.000*

PI: plaque index, GBI: gingival bleeding index, PPD: probing pocket depth, CAL: clinical attachment level. * Statistically significant (*P* ≤ 0.05).

## Discussion

 Wnt/-catenin signaling is essential for maintaining bone mass. Canonical Wnt signaling enhances osteoblast differentiation while suppressing osteoclast differentiation, thus contributing to skeletal development.^25^ Inhibitors of the Wnt signaling pathway include members of dickkopf (DKK) and secreted frizzled member protein family, Wnt modulator in surface ectoderm, Wnt inhibitory factor 1, cerebrus, and sclerostin.^17,26,27^

 Sclerostin is a bone mass-regulating glycoprotein.^8^ It acts as a bone formation suppressor.^10^ Sclerostin is a powerful inhibitor of the Wnt signaling pathway. It competitively binds to LRP5/6, preventing Wnt binding, therefore inhibiting the Canonical Wnt signaling.^14,16^ Sclerostin, a member of the differential screening-selected gene aberrative in the neuro-blastoma (DAN) family of proteins, was formerly thought to be a BMP signaling antagonist. However, recent research has identified sclerostin as a poor BMP antagonist; thus, BMP signaling is no longer regarded as a primary sclerostin activity.^13^ BMP-stimulated alkaline phosphatase and BMP/Smad signaling have also been reported to be inhibited by sclerostin.^13^ Sclerostin is an osteoblast differentiation inhibitory factor.^28^ It restricts osteoblastogenesis and downregulates the mineralization of osteoblasts.^9,12^ It also stimulates apoptosis of osteoblasts.^29^ SOST can induce the receptor activator of nuclear factor kappa-β ligand (RANKL) expression, which leads to osteoclast differentiation and bone resorption.^30^ As a result, sclerostin has both anti-osteoblastic and pro-osteoclastic activity.^1^ In vitro and in vivo studies have reported that the expression of antagonists of the Wnt/β-catenin pathway, like sclerostin, is increased in periodontitis and peri-implantitis.^1,2,11,17-20,26,31^

 This study revealed that the GCF sclerostin levels are higher in patients with SIII-GC and SIII-GB than in individuals with gingival health, consistent with the findings of Chatzopoulos et al,^1^ Rezaei Esfahrood et al,^11^ Balli et al,^2^ and Dheeraj et al.^19^ Yakar et al^20^ demonstrated that the GCF sclerostin levels between the periodontitis group and healthy participants had no statistically significant difference. However, a slight increase in the GCF levels of sclerostin was observed in patients with periodontitis.Patients with periodontitis had increased levels of SOST expression in their gingival tissue samples compared to healthy individuals.^17,18^

 Alterations in the Wnt signaling pathway or the expression of Wnt agonists and antagonists have been found to affect osteoblast development in the presence of inflammation.^27,32^ As a chronic inflammatory illness, periodontitis is characterized by elevated levels of pro-inflammatory cytokines. These inflammatory cytokines are important regulators of the Wnt pathway because they establish a positive feedback loop that regulates bone mass.^6,17^ According to recent findings, when tumor necrosis factor (TNF)-α and RANKL stimulate sclerostin synthesis, bone resorption increases, and bone formation decreases.^17,28,32,33^ Thus, the combined effect of sclerostin’s anti-anabolic function and its enhanced expression during inflammation contributes to bone resorption in patients with periodontitis.^34^

 The current investigation found that patients with SIII-GC and SIII-GB had similar GCF levels of sclerostin. To the best of our knowledge, this is the first clinical investigation to compare the GCF levels of sclerostin in patients with two different grades of periodontitis. Hence, no published literature is available to support the results of the present study. The possible reason for the insignificance in sclerostin levels between SIII-GC and SIII-GB could be that, even though the rate of progression of bone destruction between the two grades of periodontitis are different, the mechanism of bone destruction involving the Wnt signaling pathway are similar. Another reason for the insignificance is the collection of GCF samples from the deepest pocket with maximum bone degradation in both groups. Hence, the expression of sclerostin was found to be similar in both types of periodontitis.

 SOST-knockout (KO) mice exhibited higher rates of bone production, higher bone mass, more compact bone composition, and stronger bones.^35^ It has also been observed that SOST-KO mice have limited alveolar bone resorption, reduced RANKL expression, elevated osteoprotegerin (OPG) expression, and better healing of periodontal ligament and bone abnormalities.^6^

 Sclerostin can be antagonized pharmacologically using a sclerostin-neutralizing monoclonal antibody (Scl-Ab). It has been identified as a promising osteoanabolic therapy.^34^ Taut et al^36^ found that Scl-Ab promotes physiologic and therapeutic anabolic effects on alveolar bone in experimentally induced periodontitis. Yu et al^37^ demonstrated that osseointegration and bone regeneration around dental implants improved upon systemic administration of Scl-Ab.

## Conclusion

 The present investigation revealed that the GCF levels of sclerostin were upregulated in patients with SIII-GC and SIII-GB as opposed to gingivally healthy participants, confirming that it could be regarded as a potential biomarker of disease activity.

## Acknowledgments

 We would like to thank the Department of Biochemistry and the Faculty of Dentistry for supporting this study. We would also like to thank our participants for their enrolment in the study.

## Availability of Data

 The datasets used and/or analyzed during the current study are available from the corresponding author upon reasonable request.

## Competing Interests

 The authors declare that they have no financial and non-financial competing interests with regard to the publication of their work during submission.

## Ethical Approval

 Ethical approval was sought from the Institutional Ethical Committee of Rajah Muthiah Medical College and Hospital, Annamalai University (IHEC/575/2019).

## Funding

 This research received no specific grant from funding agencies in the public, commercial, or not-for-profit sectors.
